# Suicide attempt management among Turkish and American adolescents: A comparison of two pediatric emergency departments

**DOI:** 10.55730/1300-0144.5757

**Published:** 2023-11-11

**Authors:** Ali YURTSEVEN, Caner TURAN, Deborah Mary ORT, Mehrin ISLAM, Sezen KÖSE, Eylem Ulaş SAZ, Halim HENNES

**Affiliations:** 1 Department of Pediatrics, Faculty of Medicine, Ege University, İzmir, Turkiye; 2 Department of Pediatric Emergency, Southwestern Medical Center, University of Texas, Dallas, Texas, USA; 3 Department of Child and Adolescent Psychiatry, Faculty of Medicine, Ege University, İzmir, Turkiye

**Keywords:** Adolescent, cross-cultural, emergency department, suicide

## Abstract

**Background/aim:**

Suicide is one of the leading causes of death among adolescents. This study aimed to compare the characteristics and short-term outcomes of Turkish and American adolescents with suicide attempts and determine the differences in management and resource utilization between two pediatric emergency departments; one in Türkiye and one in the United States of America.

**Materials and methods:**

Adolescents who presented to the emergency departments with a chief complaint of suicide attempt between October 2017 and September 2018 were eligible for including in the study. Characteristics and other information of 217 (131 American and 86 Turkish) suicide attempter adolescents were retrieved from medical records. Outcome was defined as re-admission to the emergency department for another suicide attempt within 3 months of the index visit.

**Results:**

Overall, 78% of adolescents were female. Abuse history (physical/sexual) was more common among American adolescents (p = 0.005), whereas uncontrolled psychiatric diseases were more evident in Turkish cases (p < 0.001). Social worker assessment and hospitalization rates were significantly lower, with shorter mean duration of follow-up in the emergency department among Turkish compared to American adolescents (respectively, p < 0.001, p < 0.001 and p = 0.002). Repeated suicide attempts within three months were significantly higher in the Turkish group compared to the American one (29% vs. 8%, p < 0.001). Receiving a social worker assessment, hospitalization and longer observation in emergency department reduced the incidence of repeated suicide attempts (respectively, p < 0.001, p = 0.003 and p = 0.012).

**Conclusion:**

Turkish adolescents had shorter observation time in the emergency department, received fewer assessment by social workers and were less likely to be hospitalized. These may have contributed to the higher rate of repeat suicide attempts following discharge from the emergency department. Adequate resources are needed to help decrease the burden of suicide among Turkish adolescents.

## 1. Introduction

Suicide attempt is an act that an individual knowingly and deliberately performs with the intent of causing self-harm and/or death [1]. According to World Health Organization data, approximately 800,000 people die each year from suicide and young people age 15–29 represent more than one third of these deaths [2,3]. Worldwide, suicide is the thirteenth leading cause of deaths in all age groups and the second leading cause of deaths in adolescents and young adults [3]. The number of suicide attempts is almost 20 times higher than the number of actual deaths due to suicide [3].

In the United States of America (USA) approximately 37,000 people die from suicide every year, representing 1.6% of all deaths, with an annual suicide rate of 13/100,000 [4]. Suicide is the third leading cause of deaths among all children and adolescents in the United States [1,4]. In Türkiye, the suicide rate appears lower compared to other countries, with an annual suicide rate of 4/100,000, representing 0.8% of all deaths, however this rate tends to increase [5,6].

Most adolescents with suicide attempts present to the emergency departments (EDs) for medical care [7–9]. The ED approach to adolescents presenting with suicide attempts requires a multidisciplinary team approach to optimize patient outcome. This includes consultations with a child/adolescent psychiatrist, social worker and psychologist [3,9]. Following medical stabilization in the ED further care should be provided in a specially designated area away from the general patient care area for at least 24 h. This approach is highly recommended to prevent from repeat suicide attempts [10].

The evaluation and management of adolescents with suicide attempt should be standardized to optimize outcome [8–9]. However, such recommendation may be difficult to generalize due to variability in available resources, pediatric psychiatrists, pediatric inpatient psychiatry facilities, social workers, and counseling between institutions [10–11]. Such variability may impact patients’ subsequent suicidal ideation, long-term outcomes and prognosis.

Studies comparing adolescent suicide between developed and developing countries are limited and most of the published literature focused on epidemiologic differences [6,11,12]. In general, adolescents who live in developed countries have more suicide ideation and closer family relationships compared to those from developing countries counterparts, whereas , adolescents who live in developing countries are more religious and exposed to more negative attitudes about suicide [6,11,12]. Although some characteristics of these two groups are different, predisposing factors that lead them to suicide and the appropriate treatment approaches may be similar to each other [3,9,11]. However, data comparing ED management and its impact on the short-term outcome of these patients between institutions with different resources is unavailable.

This study objective is to compare the evaluation, management, resource utilization and short-term outcome (repeat suicide attempt within 3 months following the index visit) of adolescents who present to the ED with a chief complaint of suicide attempt at two different tertiary care institutions in two countries and identify opportunities for improving the care and outcome of adolescents with suicide attempts in Türkiye.

## 2. Materials and methods

### 2.1. Study design and population

This is a retrospective observational study conducted at two tertiary care pediatric hospitals EDs, one in the USA, Dallas (The University of Texas Southwestern Medical Center) and one in Türkiye, İzmir (Ege University Faculty of Medicine). The University of Texas Southwestern Medical Center, Pediatric ED is one of the busiest pediatric EDs in the USA with more than 120,000 annual visits. Stable patients with a behavioral health related chief complaint receive care in a designated area of the ED. Ege University Faculty of Medicine, Children’s Hospital is one of the largest tertiary medical center in Türkiye with more than 90,000 visits the pediatric ED annually. All adolescents (aged 13–18) who presented to both EDs with suicide attempts between October 2017 and September 2018 were identified from the hospital and ED database. Demographic information, chief complaint, presenting symptoms, clinical findings, outcome and utilized resources were abstracted from the electronic medical records.

### 2.2. Management of suicide attempt

At both centers, an approach based on the recommendations of the American Academy of Pediatrics Committee on Adolescence is applied from the prestudy period. According to this approach the adolescents who present to the EDs due to suicide attempts are divided by a child/adolescent psychiatrist into three groups, as mild-, moderate- and high-risk as follows [1].

Mild risk: Taking harmless types or quantities of medication after a simple argument, informing relatives shortly after drug intake, absence of a serious problem at home and/or school, absence of depression or any other severe psychiatric disease, having a close friend, desiring to solve existing problems , and suicide attempt shortly after an argument.

Moderate risk: Recurring arguments with the family, poor school achievement, wishing to leave the family, wrecking and destroying the environment while alone, tearing up things they have written, leaving the family, changing school, presence of attention deficit hyperactivity disorder, recent exhibition of depressive characteristics, inability to control anger, drinking large quantities of alcohol at least twice a week, agreeing to speak to a specialist after a suicide attempt and responding all questions during the interview.

High risk: Exposure to a severe family reaction due to narcotic use, recently breaking up with a girl/boyfriend, the recent death of a close friend, wishing to die from a belief that life is pointless, taking a gun from home and shooting or attempting to shoot oneself saying there is no other way out, frequent alcohol and narcotics use, frequent running away from home or playing truant from school saying nobody likes them, admission to hospital due to loss of consciousness, refusing to respond to questions during the interview or overreacting to them.

All adolescents included in the study were managed accordingly to their risk assessment status.

### 2.3. Measures

Patient demographic, social and clinical characteristics (age, sex, school attendance, living with whom, type of suicide attempt, location of attempt, current stress factor, prior suicide attempt, history of psychiatric illness, regular psychiatrist follow-up , and having a life-threatening condition), parental characteristics (marital status, domestic lifestyle, their reaction to the suicide attempt, history of psychiatric disease and suicide) and patient care and assessment characteristics in the ED (after medical stabilization followed-up in a separate area in the ED, psychiatrist assessment, psychologist assessment, psychiatric nurse assessment, social worker assessment, hospitalization rate, duration of observation of discharged patients in the ED) were abstracted and entered into study database.

Patients readmitted to the ED with repeat suicide attempt within 3 months following the index visit were identified from the database and their medical records reviewed and data abstracted.

### 2.4. Data analysis

Data were analyzed using Statistical Package for the Social Sciences for Windows 21.0 software. Descriptive statistical were expressed as mean ± standard deviation for continuous variables and percentage for categorical variables. Chi-square test was used to compare categorical data, t test and one-way ANOVA were used to compare measurement values. P–values of <0.05 were regarded as statistically significant. Odds ratio and confidence interval were calculated when appropriate.

## 3. Results

During the study period, 131 American and 86 Turkish adolescents presented to the EDs with a suicide attempt. The rates of suicide attempts were close in both centers according to the total number of admissions. Based on current stress factors; physical/sexual abuse (p = 0.005, OR: 3, 95% CI: 1.4–4.8) and peer bullying (p = 0.039, OR: 2.7, 95% CI: 1.3–4.4) were significantly more evident in the American adolescents than the Turkish. The presence of psychiatric illness, on-going psychiatric care and previous suicide attempts were significantly higher in the American group compared to the Turkish one (respectively, p = 0.006, OR: 2.8, 95% CI: 1.4–5.8, p < 0.001, OR: 3.1, 95% CI. 1.7–5.6, and p = 0.001, OR: 2.7, 95% CI: 1.5–4.8). No difference was found in other demographic and clinical characteristics (Table 1).

Family history of psychiatric disease and the proportion of parents who are either divorced or separated were significantly higher in the American group compared to the Turkish group (respectively, p = 0.037, OR: 1.8, 95% CI: 1.1–3.2 and p = 0.003, OR: 2.3, 95% CI: 1.3–4.1). Parents of the American group were more supportive and compassionate towards their children whereas parents of the Turkish group were angry (p = 0.014, OR: 2.3, 95% CI: 1.2–4). The other parental characteristics of both groups are summarized in Table 2.

In the American group a social worker assessment and hospitalization rate were significantly higher, the mean length of stay in the ED was longer than that of the Turkish group (respectively, p < 0.001, OR: 9.2, 95% CI: 5.8–14.6, p < 0.001, OR: 16.6, 95% CI: 8.3–33.3, and p = 0.002, mean difference: −12.6, 95% CI: −20 to −5). All American cases stayed on observation in a separate area in the ED following clinical stabilization, whereas none from the Turkish group had a similar opportunity. Medical care that was provided to adolescents in both EDs is shown in Table 3.

Reattempted suicide rates following ED discharge were significantly higher among Turkish adolescents compared to American patients (29% vs. 8%, p < 0.001, OR: 4.9, 95% CI: 2.2–10.9). In reattempted suicide group, the rates of social worker evaluation and hospitalization were lower and the length of ED stay was shorter (respectively, p < 0.001, OR: 4, 95% CI: 1.9–8.6, p = 0.003, OR: 3.3, 95% CI: 1.5–7.2, and p = 0.012, mean difference: −6.2; 95% CI: −11.5 to −1). Associated factors with suicide reattempt within 3 months after discharge are shown in Table 4. Three Turkish adolescents died (3/86) as a result of suicide attempts and there were no mortalities in the American study population.

## 4. Discussion

This study compared the evaluation and management of adolescents with suicide attempts in two tertiary care Children’s Hospitals EDs in two different countries (USA and Türkiye). Our goal for this comparison is to identify any differences in the evaluation or management of these patients and the potential effect on outcome. Most variables such as, femalesex, suicide attempt by ingesting medication, and a history of psychiatric disease was similar in both populations. These findings are consistent with the results reported in previously published studies [13–15]. However, observing a higher rate of repeat suicide attempts among Turkish adolescents compared to adolescents in the USA was interesting. This finding may be due to the limited resources available for the Turkish population. Specifically, the lack of psychiatric social workers’ availability in the ED, availability of pediatric psychiatric support and in-patient pediatric psychiatric facilities. To further validate our observation a larger prospective multicenter observation study may be necessary.

Factors contributing to the increased risk of suicide attempts in adolescents are well documented in the literature [6–9]. These include; history of psychiatric disorder, family problems, history of physical/sexual abuse, prior suicide attempts and peer bullying. These predisposing factors play a crucial role in driving adolescents to attempt suicide [15–18]. In reviewing the medical records of the study population these risk factors were identified among both study populations.

Parental characteristics and parent–child relationships should be asked in adolescents presenting with a suicide attempt. Strained parental relationships and/or family history of psychiatric illness also contributed to adolescents’ suicide attempts [19,20]. Impaired parent–child relationships, lack of family support and parental mistreatment during adolescence years have been shown to markedly increase the risk of adolescent suicide attempts [21]. Similarly, these characteristics were observed in most parents of the both groups in our study.

Studies have shown that an appropriate ED approach to adolescents with suicide attempts can improve medication compliance and decrease the risk of future suicide attempts [3,20–24]. In our cohort, both groups were assessed by a child and adolescent psychiatrist in the ED. Psychiatric social worker assessment was done for all American adolescents and they were observed in a designated area following clinical stabilization in the ED and the majority were hospitalized following the ED management. The availability of a dedicated area in the ED allowed for longer observation time in the ED for low risk patients and an opportunity to plan for safe discharge and plans for follow up. However, only a small number of Turkish adolescents were assessed by a social worker and/or hospitalized and none of them were observed in a separate area in the ED. The gap of economic opportunities and healthcare quality between the two countries most likely explain the difference.^1^

^1^World Population Review (2023). CEOWORLD Health Care Index, 2023 [online]. Website https://worldpopulationreview.com/country-rankings/best-healthcare-in-the-world [accessed 7 June 2023].

In Türkiye, the burden of care for these patients falls on the ED staff who have limited training in psychiatric disorders evaluation and cannot provide the time needed for counseling patients and families or assist them finding appropriate community resources in Türkiye. Also, Türkiye as a Middle Eastern country, perspectives of Turkish families about mental illness may hinder seeking psychiatric help. These problems may have affected the treatment and follow-up of Turkish adolescents resulting in higher rate of reattempted suicide.

Repetition of suicide attempts is common in adolescents [25–27]. Predisposing factors leading to suicidal behavior must be determined to reduce the risk of suicide reattempt [26–28]. As these patients generally seek care in the EDs following, the EDs need to have the resources available to provide adequate and safe care for these patients and optimize their outcome. In this study, we only assessed the suicide reattempt rate within 3 months of the index visit and it is possible that a longer denaturation may have yielded different rates than wheat we reported. However, suicide reattempt and mortality rates among the Turkish population is unacceptably high as we explained above. Steps should be taken to allocate more resources and explore opportunities for improving the care of this population.

This study has several limitations. First, this is a retrospective study. Hence these findings can be over or underestimated. Second, it reflects the experience of a single center; therefore, its results cannot be generalizable to other settings. Third, some features of the cases were obtained from the cases or their families during the follow-up, thus, some information of the study may be incorrect. Fourth, the cases included in the study were relatively unsatisfactory. This low number may result in a less representative sample. Another limitation is that this study is a cross-cultural research. The data was obtained by researchers from different cultures in different centers. Different perceptions of the researchers may affect the result of the study. Despite these limitations, this is the first study that compares the ED management and its impact on the short-term outcome of Turkish and American adolescents with suicide attempts and determines the deficiencies of health care offered to this cases.

In conclusion, there was a difference in the demographics, family dynamics, ED evaluation and management of adolescents who presented to the ED with suicide attempts between the two study cohorts. Turkish adolescents had shorter observation time in the ED, received fewer assessments by social workers and were less likely to be hospitalized. Even though these problems did not directly cause suicide attempts, they may have contributed to the higher rate of repeat suicide attempts following discharge from the ED. Specially designated areas in the EDs, sufficient social workers and adequate in-patient pediatric psychiatric facilities are needed to help the prevention of repeated suicide attempts of adolescents in Türkiye.

## Figures and Tables

**Table 1:** Demographic, clinical, and social characteristics of all adolescents.

Characteristics	American (n = 131)	Turkish (n = 86)	Total (n = 217)	p-value	Effect size*

Sex (Female), n(%)	103(79)	67(78)	170(78)	0.900	1.1(0.8–1.3)

Mean age (years) (± SD)	15.2(1.4)	15.3(1.2)	15.3(1.3)	0.615	0,1(−0.2 to 0.4)

Regularly school attend, n(%)	105(80)	56(65)	161(74)	**0.017**	2.2(1.2–4)

Living with both M and F, n(%)	46(35)	48(56)	94(43)	**0.003**	2.3(1.3–4.1)

Living with step M or F, n(%)	30(23)	5(6)	35(16)	**<0.001**	4.9(2.1–8.2)

SA with drug ingestion n(%)	112(86)	74(86)	186(86)	1.000	1.0(0.5–2.3)

SA at home, n(%)	115(88)	75(87)	190(88)	0.900	1.1(0.4–2.1)

Main current stress factor, n(%)					
Domestic family issues	44(34)	21(24)	65(30)	0.173	1.6(1.1–1.9)
Uncontrolled psychiatric disease	21(16)	43(50)	64(29)	**<0.001**	5.3(2.3–8.6)
Abuse (physical/sexual)	34(26)	9(11)	43(20)	**0.005**	3.0(1.4–4.8)
Peer bullying	22(16)	6(7)	28(13)	**0.039**	2.7(1.3–4.4)

Previously SA, n(%)	88(67)	37(43)	125(58)	**0.001**	2.7(1.5–4.8)

Having any psychiatric disease, n(%)	116(89)	63(73)	179(83)	**0.006**	2.8(1.4–5.8)

Previously regular PF, n(%)	66(51)	21(24)	87(40)	**<0.001**	3.1(1.7–5.6)

Life-threatening condition, n(%)	16(12)	13(15)	29(13)	0.547	1.1(0.8–1.6)

M: Mother, F: Father, PF: Psychiatrist follow-up, SA: Suicide attempt,

* For categorical data by odds ratio, for continuous data by mean difference with 95% confidence intervals.

**Table 2:** Parental characteristics of all adolescents.

Characteristics	American (n = 131)	Turkish (n = 86)	Total (n = 217)	p-value	OR (95% CI)

Marital status of parents, n(%)				**0.003**	2.3(1.3–4.1)
Married and living together	46(35)	48(56)	94(43)		
Divorced and/or Separated	85(65)	38(44)	123(57)		

Relationship between parents, n(%)				**0.001**	2.8(1.4–4.8)
Regular/Good	25(19)	34(40)	59(27)		
Strained/No relationship	106(81)	52(60)	158(73)		

Having any PD, n(%)				**0.037**	1.8(1.1–3.2)
Yes	73(56)	35(41)	108(50)		
No/Unknown	58(44)	51(59)	109(50)		

Having any SH, n(%)				0.280	1.5(0.7–3.2)
Yes	26(20)	12(14)	38(18)		
No/Unknown	105(80)	74(86)	179(82)		

Attitude to the event, n(%)				**0.014**	2.3(1.2–4.0)
Concern/Supportive	107(82)	57(66)	164(76)		
Angry/Unknown	24(18)	29(34)	63(24)		

PD: Psychiatric disease, SH: Suicide history, OR: Odds ratio, CI: Confidence intervals

**Table 3:** The comparison of medical care provided to adolescents in both centers.

Characteristics	American (n = 131)	Turkish (n = 86)	Total (n = 217)	P Value	Effect size*

CAP assessment (+), n(%)	131(100)	83(97)	214(99)	0.061	1.1(1.0–1.3)

Social worker assessment (+), n(%)	131(100)	17(20)	148(68)	**<0.001**	9.2(5.8–14.6)

How were patients concluded, n(%)				**<0.001**	16.6(8.3–33.3)
Discharged from the ED	29(22)	71(83)	100(46)		
Admission to the hospital	102(78)	15(17)	117(54)		

Mean duration of follow-up of NHP in the ED, (hours) (± SD)	21.9(17.4)	9.3(4.7)	13.3(10.9)	**0.002**	−12.6 (−20 to −5)

CAP: Child and adolescent psychiatrist, NHP: Nonhospitalized patient, ED: Emergency department,

* For categorical data by odds ratio, for continuous data by mean difference with 95% confidence intervals

**Table 4:** Factors associated with suicide re-attempt within 3 months after discharge.

Characteristics	Suicide reattempt (+) 34(16%)	Suicide reattempt (−) 180(84%)	p-value	Effect size*

Sex (Female), n(%)	26(15)	144(85)	0.646	1.2(0.5–2.9)

Orgin, n(%)			**<0.001**	4.9(2.2–10.9)
American	10(8)	121(92)		
Turkish	24(29)	59(71)		

Living with both M and F, n(%)	17(50)	75(42)	0.450	1.4(0.7–3.1)

Previous suicide attempt (+), n(%)	25(74)	98(54)	0.057	2.3(1.1–5.3)

Having any psychiatric disease, n(%)	30(88)	147(82)	0.462	1.7(0.6–5.1)

Previously regular PF (+), n(%)	15(44)	71(39)	0.704	1.2(0.6–2.5)

Marital status of the parents, n(%)			0.451	1.4(0.7–2.9)
Married and living together	17(50)	75(42)		
Divorced and/or Separated	17(50)	105(58)		

Relationship between parents, n(%)			0.676	1.2(0.7–2.4)
Regular/Good	10(29)	47(35)		
Strained/No relationship	24(71)	133(65)		

SW assessment in the ED (+), n(%)	14(41)	133(74)	**<0.001**	4(1.9–8.6)

How were patients concluded, n(%)			**0.003**	3.3(1.5–7.2)
Discharged from the ED	24(71)	76(42)		
Hospital admission	10(29)	104(58)		

Mean duration of follow-up of NHP in the ED, (hours) (± SD)	13.3(10.7)	20(16.9)	**0.012**	−6.2(−11.5 to −1)

ED: Emergency department, F: Father, M: Mother, NHP: Nonhospitalized patients, PF: Psychiatrist follow-up, SW: Social worker,

* For categorical data by odds ratio, for continuous data by mean difference with 95% confidence intervals
